# Supplementary material for: Nanopore direct RNA sequencing maps the complexity of Arabidopsis mRNA processing and m6A modification
Source: eLife. 2020 Jan 14;9:e49658. doi: 10.7554/eLife.49658 (PMC6959997; doi:10.7554/eLife.49658)
Supplement: Transparent reporting form [file elife-49658-transrepform.docx]

***eLife’s* transparent reporting form**

We encourage authors to provide detailed information *within their submission* to facilitate the interpretation and replication of experiments. Authors can upload supporting documentation to indicate the use of appropriate reporting guidelines for health-related research (see [EQUATOR Network](http://www.equator-network.org/%20)), life science research (see the [BioSharing Information Resource](https://biosharing.org/)), or the [ARRIVE guidelines](http://www.plosbiology.org/article/info:doi/10.1371/journal.pbio.1000412) for reporting work involving animal research. Where applicable, authors should refer to any relevant reporting standards documents in this form.

If you have any questions, please consult our Journal Policies and/or contact us: [editorial@elifesciences.org](mailto:editorial@elifesciences.org).

**Sample-size estimation**

- You should state whether an appropriate sample size was computed when the study was being designed
- You should state the statistical method of sample size computation and any required assumptions
- If no explicit power analysis was used, you should describe how you decided what sample (replicate) size (number) to use

Please outline where this information can be found within the submission (e.g., sections or figure legends), or explain why this information doesn’t apply to your submission:

No explicit power analysis was used. For the sequencing experiments, we had previously analyzed the properties of Illumina RNA-Seq data (Schurch et al., [2016] How many biological replicates are needed in an RNA-seq experiment and which differential expression tool should you use? ***RNA*** **22**: 839-51). Consequently, we followed the recommendations of this study and used at least 6 biological replicates for each RNASeq dataset here (Supplemental table 2). However, no equivalent analysis of the properties of either miCLIP or nanopore DRS has yet been performed (to our knowledge). Consequently, for nanopore DRS we used 4 biological replicates to enable greater coverage for Col-0 analysis and statistical power for *vir* versus VIRc analyses (Supplemental table 1). The ENCODE guidance for eCLIP, which is related, in part to miCLIP, suggests that the use of at least two biological replicates to allow for irreproducible discovery rate analysis. We therefore used three biological replicates of paired miCLIP and input RNA-seq data. One replicate of miCLIP was omitted from the analysis due to lower reproducibility. For RT-qPCR we used three biological replicates and three technical replicates of each biological condition as this is a well-accepted approach in molecular biology. We used three biological replicates for the LC-MS analysis of m6A according to previous analyses that established this approach (reference cited in Materials and methods). Delayed fluorescence measurements were recorded over a time-course for at least 20 technical replicates of each genotype. The entire experiment was repeated twice and although only one dataset is included in Figure 6B and C, both datasets are available in supplementary dataset 16. The use of such sample sizes was established by the author Anthony Hall and the corresponding reference is cited in the Materials and methods section.

**Replicates**

- You should report how often each experiment was performed
- You should include a definition of biological versus technical replication
- The data obtained should be provided and sufficient information should be provided to indicate the number of independent biological and/or technical replicates
- If you encountered any outliers, you should describe how these were handled
- Criteria for exclusion/inclusion of data should be clearly stated
- High-throughput sequence data should be uploaded before submission, with a private link for reviewers provided (these are available from both GEO and ArrayExpress)

Please outline where this information can be found within the submission (e.g., sections or figure legends), or explain why this information doesn’t apply to your submission:

Biological replicates for Illumina and Nanopore sequencing are stated in the main text. All samples sequenced for nanopore are also stated in Table S1. Technical replicates were not conducted, except for a re-run of Col-0 bio replicate 2 of Nanopore DRS, which was re-run due to a software error during sequencing. This is stated in Table S1.

Sequencing data will be made available through ENA, accession number is stated in Data Availability section.

**Statistical reporting**

- Statistical analysis methods should be described and justified
- Raw data should be presented in figures whenever informative to do so (typically when N per group is less than 10)
- For each experiment, you should identify the statistical tests used, exact values of N, definitions of center, methods of multiple test correction, and dispersion and precision measures (e.g., mean, median, SD, SEM, confidence intervals; and, for the major substantive results, a measure of effect size (e.g., Pearson's r, Cohen's d)
- Report exact p-values wherever possible alongside the summary statistics and 95% confidence intervals. These should be reported for all key questions and not only when the p-value is less than 0.05.

Please outline where this information can be found within the submission (e.g., sections or figure legends), or explain why this information doesn’t apply to your submission:

Statistical analysis methods are outlined in Materials and methods.

Raw data is presented as overlayed stripcharts for boxplots of qPCR, single gene RNAseq, and period length experiments.

N values for histograms are either labelled on figures or stated in the legend.

P values and 95% confidence intervals generated by bootstrapping are reported in the main text.

(For large datasets, or papers with a very large number of statistical tests, you may upload a single table file with tests, Ns, etc., with reference to sections in the manuscript.)

**Group allocation**

- Indicate how samples were allocated into experimental groups (in the case of clinical studies, please specify allocation to treatment method); if randomization was used, please also state if restricted randomization was applied
- Indicate if masking was used during group allocation, data collection and/or data analysis

Please outline where this information can be found within the submission (e.g., sections or figure legends), or explain why this information doesn’t apply to your submission:

Samples were assigned to groups based on genotype, no treatments were applied.

Blinding was not used for data collection or analysis.

**Additional data files (“source data”)**

- We encourage you to upload relevant additional data files, such as numerical data that are represented as a graph in a figure, or as a summary table
- Where provided, these should be in the most useful format, and they can be uploaded as “Source data” files linked to a main figure or table
- Include model definition files including the full list of parameters used
- Include code used for data analysis (e.g., R, MatLab)
- Avoid stating that data files are “available upon request”

Please indicate the figures or tables for which source data files have been provided:

All source code will be made available on GitHub as stated in the Materials and methods section.

Supplemental datasets for the following figures are provided:

Figure 1C, S1G: sd01_hen2-2_vs_col0_illumina_DERfinder.bed.tds

Figure S2A: sd02_ercc_polya_lengths.tsv.gz.zip

Figure 2A: sd03_col0_polya_lengths.tsv.gz.zip

Figure S2B: sd04_col0_per_gene_polya_dists.tsds

Figure 3C, 3E, S3G: sd05_riken_tss.bed.tds

Figure 3C, 3E, S3G: sd06_nanoPARE_peaks.bed.tds

Figure S3H: sd07_nanopore_mirna_cleavage.xlsx

Figure S4A: sd08_splice_junction_sanger_sequencing_products.fa.txt

Figure S5A: sd09_MALAT1_differential_error_sites.bed.tds

Figure S5B: sd10_lcms_m6a_content.xlsx

Figure 5-7: sd11_vir1_vs_VIRc_nanopore_differential_error_sites.bed.tds

Figure 5B, S5C: sd12_vir1_vs_VIRc_differential_error_motifs_unique.gtf.tds

Figure 5E, 5F, S5D: sd13_miCLIP_peaks.bed.tds

Figure 6A: sd14_vir1_vs_VIRc_nanopore_differential_expression.xlsx

Figure S6A: sd15_vir1_vs_VIRc_illumina_differential_expression.xlsx

Figure 6B, 6C: sd16_delayed_fluorescence_results.xlsx

Figure 6D: sd17_vir1_vs_VIRc_differential_polya_dists.tds

Figure 6D: sd18_cab1_polya_dists.tds

Figure S6C, S6D: sd19_cca1_flc_qpcr_data.xlsx

Figure S7A: sd20_vir1_vs_VIRc_illumina_differential_exon_usage.xlsx

Figure 7A: sd21_vir1_vs_VIRc_nanopore_differential_polya_site.xlsx

Figure S7B: sd22_vir1_vs_VIR_GFP_dapars_results.tds

Figure 7F: sd23_vir1_vs_VIRc_illumina_DERfinder.bed.tds
